# Assessment by ABPM verified the presence of hypertension in patients with self-reported hypertension, pregnant women, as well as differences between ethnicities in women aged 38-39 years in the Ribeirão Preto cohort

**DOI:** 10.3389/fphar.2022.992595

**Published:** 2022-11-09

**Authors:** Tetzi Oliveira Brandão, Eduardo Carvalho de Arruda Veiga, Rozeli Ferreira Levy, Enio Luis Damaso, Valeria Cristina Sandrim, Ricardo Carvalho Cavalli

**Affiliations:** ^1^ Department of Gynecology and Obstetrics, Ribeirao Preto Medical School, University of Sao Paulo, Sao Paulo, Brazil; ^2^ Department of Physiotherapy, Federal University of Paraiba, Sao Paulo, Brazil; ^3^ Department of Pediatric Dentistry, Orthodontics and Public Health, Bauru School of Dentistry, University of São Paulo, Sao Paulo, Brazil; ^4^ Department of Pharmacology and Biophysics, Institute of Biosciences, Sao Paulo State University (Unesp), São Paulo, Brazil

**Keywords:** ambulatory blood pressure monitoring, comorbidities, hypertension during pregnancy, blood pressure, hypertension

## Abstract

**Introduction:** Arterial hypertension is a global health problem and one of the main risk factors for cardiovascular diseases (CVD), and therefore for morbidity and mortality among adult men and women. Factors related to obstetric history, family history, sociodemographic characteristics, and lifestyle habits are known determinants of arterial hypertension.

**Methods:** Case-control study of women belonging to the 1978/79 birth cohort conducted in the city of Ribeirão Preto/SP. Sociodemographic data, presence of comorbidities, maternal comorbidities, paternal comorbidities, comorbidities during pregnancy, and biometric and biophysical markers associated with blood pressure measured by 24-h ambulatory blood pressure monitoring (ABPM) were assessed in women aged 38–39 years. We want to study which variables of the previous sentence are related to the presence of hypertension measured by ABPM.

**Results:** Data from 281 women were analyzed. Our results showed that ethnicity, a history of hypertension, and gestational hypertension reported by the women were significantly associated with the presence of hypertension measured by ABPM. Other factors such as marital status, educational level, comorbidities of the woman, paternal or maternal comorbidities, anthropometric measurements or serum levels of cardiovascular markers were not associated with the presence of hypertension measured by ABPM.

**Conclusion**: We conclude that ethnicity, self-reported hypertension, and gestational hypertension are associated with arterial hypertension measured by ABPM.

## Introduction

Birth cohort studies have been high priority on the research agenda of developed countries for scientific and technological advancement (Brandstetter et al., 2019). Arterial hypertension is a chronic disease and a public health problem in Brazil and in the world. In addition, it is one of the main risk factors for cardiovascular diseases (CVD) and a leading cause of death worldwide (Brant et al., 2022) (Ribeiro et al., 2016) Arterial hypertension is characterized by persistently raised blood pressure, i.e., systolic blood pressure (SBP) ≥ 130 mmHg and/or diastolic blood pressure (DBP) ≥ 80 mmHg, measured with the correct technique on three different occasions in the absence of antihypertensive medication (Whelton et al., 2018).

After pregnancy, women with hypertensive disorders of pregnancy such as gestational hypertension and preeclampsia are at increased risk of developing CVD (Honigberg and Natarajan, 2020). Preeclampsia is a specific pregnancy syndrome that affects about 5%–8% of pregnancies and is a leading cause of maternal, fetal and neonatal mortality. The condition is characterized by raised blood pressure after the 20th week of gestation (Rana et al., 2019).

Metabolic syndrome is defined by the health word organization (WHO) as a cluster of conditions that include arterial hypertension, abdominal obesity, dyslipidemia, and altered glucose metabolism (Fahed et al., 2022). It is estimated that about one quarter of the world’s population has metabolic syndrome, corresponding to more than one billion people. In Brazil, a prevalence of metabolic syndrome in the adult population of 29.6% has been reported, which can reach more than 40% in age groups over 60 years (Oliveira et al., 2020; de Siqueira Valadares et al., 2022).

Studies of blood pressure measurements are uncommon in the Brazilian population and most existing ones are restricted to certain locations and using different information, a fact impairing comparison of the data (Jardim et al., 2020). In addition, different diagnostic criteria exist for estimating the population prevalence of arterial hypertension, such as cut-off point and the use or not of associated medications (Zhou et al., 2021)

Arterial hypertension is a chronic disease whose incidence is increasing worldwide and that is reported to be one of the main causes of CVD, with a consequent impact on mortality, morbidity, and quality of life (Mills et al., 2020). Knowledge of the most important and prevalent risk factors related to the pathophysiology of this disease in women will permit to prevent this condition by proposing early interventions and more appropriate treatments (Carey et al., 2018). Our hypothesis was that with the technique measured by ambulatory blood pressure monitoring the study would be able to identify hypertension in the woman’s gestational phase, in self-reported hypertension and other variables in women aged close to 40 years.

## Materials and methods

### Study design

Analytical, retrospective, case-control study that included female patients of the Ribeirão Preto Cohort 1978/1979. The 1978/79 cohort, as the group of participants is called, is the first National Cohort in Brazil and has 6827 live births in the city of Ribeirão Preto. In addition to the assessment at birth, these individuals were also assessed at school age, at military enlistment, at 23–25 years, and in their 5th assessment (2016/2017), in adulthood (38–39 years), in which 1775 were evaluated. Individuals being 929 women. Reinforcing the data of the participants of this study were carried out on women who were aged between 38 and 39 years. These women were invited to attend spontaneously interviews and examinations at the University of São Paulo, at the Hospital das Clínicas of the Faculty of Medicine of Ribeirão Preto. Of the 929 patients who attended, 281 were accepted to have the ambulatorial blood pressure monitoring (ABPM) placed. The placement of the ABPM device was an option for the participants and not a criterion of the study. Measurement blood pressure was performed for 24 h from ABPM placement. ABPM analysis measures data on mean 24-h systolic and diastolic blood pressure, and mean systolic and diastolic blood pressure during wakefulness and sleep. After these data, the study´s cardiology physicians lauded the participants´ABPM and classified them as hypertensive or normotensive (de Souza et al., 2022). The inclusion criteria were female participants, not pregnant at the time of the interview and who accepted the measurement by ABPM. And the exclusion criterion was male and women who refused to be measured by ABPM.

### Evaluation of anthropometric measurements

The evaluation of anthropometric measurements was made through measurements of weight, height, calculation of Body Mass Index (BMI), waist circumference (WC), and neck circumference (NC).

### Arterial stiffness assessment

Pulse wave velocity (PWV) analysis is a simple, non-invasive, and reliable diagnostic method for the assessment of arterial stiffness. (47) The computerized system Sphygmocor^®^ Software Version 9.0, AtCor Medical Pty Ltd., was used to record and analyze PWV. The measurement of PWV was performed with the participant in the supine position after a 5-min rest and an acclimatized room (22–24°C). An inelastic measuring tape graduated in centimeters, was used to measure the distances between the carotid wrist and the sternal notch and between the sternum notch and the femoral wrist. External transducers were placed directly on the skin in the right carotid artery and the right femoral artery. Capture the recording of pulse waves for a minimum time interval of 10 s. The PWV measurement is automatically calculated by the ratio of the carotid-femoral distance and the time interval between the two pulses.

### Blood and urine collection

The participants had blood collection performed at the Gynecology Laboratory of the Hospital das Clínicas of the Faculty of Medicine of the University of São Paulo in the city of Ribeirão Preto. Blood collection was not performed on an empty stomach. From each subject, 20 ml of whole blood was collected and stored in conical plastic tubes (BD-Becton Dickinson, Plymouth, United Kingdom). The women did not use any medication that could alter the results of laboratory tests. Blood samples were processed within a maximum of 2 h after collection. The serum was stored at −80°C for measurement of all serum variables at the same time.

Serum concentrations of creatinine, total cholesterol, high-density lipoprotein, low-density lipoprotein, triglycerides, C-reactive protein, homocysteine, basal insulin, and glucose were quantified in an automated biochemistry analyzer (Weiner, Rosario, Argentina). The glomerular filtration rate was estimated from creatinine and cystatin C using the CKD-EPI 2009 equation. (15) Glycated hemoglobin (HbA1c) was determined by HPLC (Bio-Rad D-10, Hercules, CA).

### Questionnaire applied to women

The questionnaire asked about demographic variables such as skin color, marital status, social benefit, and educational level (Years of schooling). After the questionnaire, ABPM was measured, and for each demographic variable mentioned above, evaluated the presence of hypertension.

In the questionnaire, the patient was asked about the presence of comorbidities. Among the questions on the questionnaire, the patient was asked if she was aware of the presence of the following comorbidities: high blood glucose/diabetes, hypercholesterolemia, hypertension, obesity, thrombosis, arrhythmia, angina, infarction, stroke, kidney disease. After this questionnaire, the participants placed the ABPM, and the presence of hypertension measured by the ABPM was evaluated. With this, we obtained two groups about to with concerning these variables of the presence of comorbidities, the hypertensive related to these comorbidities and the normotensive related to these comorbidities.

In the questionnaire, the women were asked about the following paternal and maternal comorbidities: knowledge of the father’s disease, paternal high blood glucose/diabetes, hypercholesterolemia reported by the father, cardiovascular disease described by the father, paternal hypertension, paternal obesity, paternal thrombosis, paternal arrhythmia, paternal angina, paternal infarction, paternal stroke, paternal kidney disease, knowledge of the mother’s disease, maternal high blood glucose/diabetes, hypercholesterolemia reported by the mother, cardiovascular disease reported by the mother, maternal hypertension, maternal obesity, maternal thrombosis, maternal arrhythmia, maternal angina, maternal infarction, maternal stroke, and maternal kidney disease. And for each variable of paternal or maternal comorbidity, evaluated the presence or absence of hypertension.

Another evaluation performed was the presence of hypertension measured by MAPA in pregnant women with the following questions for women if they were aware of the following comorbidities during pregnancy: self-reported preeclampsia and gestational hypertension.

### Blood pressure measurement by ambulatory blood pressure monitoring

In this study, blood pressure was measured by ABPM. The installation of the device (A&D Company Limited, Kitamoto-shi, Saitama-ken, Japan) was offered to all women on the day of the interview but the decision to use the device was voluntary.

The patient was classified as hypertensive based on ABPM if the mean SBP ≥130 mmHg or mean DBP ≥80 mmHg (Whelton et al., 2018; Flynn et al., 2022). The obstetric history on the presence of hypertensive syndromes and current hypertension was obtained with the questionnaire during the interview and not from the medical record (Barbieri et al., 2006; Malta et al., 2018; Hurrell et al., 2022).

### Statistical analysis

Continuous and semi-continuous variables were compared using a Gauss curve. Data were found to be nonparametric by the Kolmogorov-Smirnov and Shapiro-Wilk tests and were therefore reported as median and percentiles (25–75). The geometric mean was used when the sample showed a high frequency of modal values (>20%). Two independent groups were compared by the Mann-Whitney test with Bonferroni correction. Categorical data were expressed as absolute (n) and relative (%) frequency. The contingency matrices were analyzed by Pearson’s chi-squared test and complex matrices with *p* ≤ 0.05.

## Results

Of the 929 patients of the cohort, only 281 underwent ABPM. Twenty-four-hour SBP and DBP were recorded, as well as sleeping and awake SBP and DBP, and are expressed as median and percentiles (Table 1). The median SBP was 113 mmHg and the median DBP was 72 mmHg. Blood pressures differed between the sleep and wake periods, with a median SBP and DBP of 117 and 76 mmHg during the wake period, respectively, while SBP was 106 mmHg and DBP was 65 mmHg during the sleep period.

**TABLE 1:** Description of ABPM results (*n* = 281).

	Median	25%	75%
ABPM mean 24-h SBP	113.0	108.0	120.0
ABPM mean 24-h DBP	72.0	68.0	79.0
ABPM mean awake SBP	117.0	111.0	122.0
ABPM mean awake DBP	76.0	72.0	84.0
ABPM mean sleep SBP	106.0	100.0	113.0
ABPM mean sleep DBP	65.0	60.0	71.0

Data expressed as median and percentiles. ABPM, ambulatory blood pressure monitoring; SBP, systolic blood pressure; DBP, diastolic blood pressure.

**FIGURE 1:**
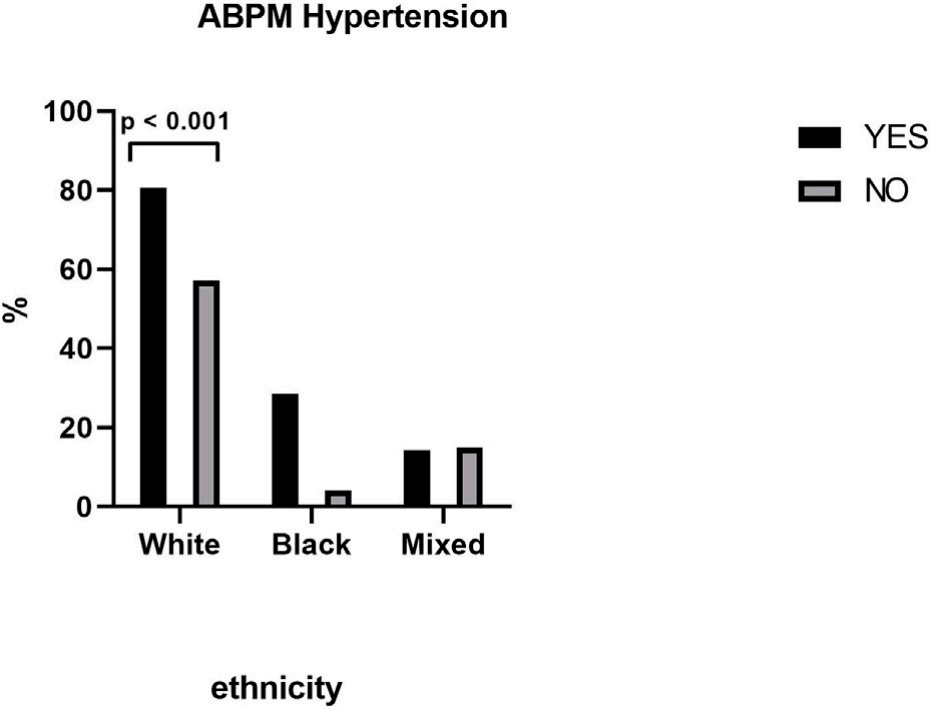
ABPM Hypertension between ethnicities.

Figure 1 shows the demographic variable of the e. A significant difference (*p* = 0.001) was only observed for ethnicity, with a higher prevalence of hypertensive women among white patients. In Table 2 there was no difference in marital status, i.e., if the women were married or lived with a partner, if they were separated or divorced, or if they were single or widowed, concluding that marital status does not influence the presence or absence of hypertension measured by ABPM. Receiving social benefit or educational level also did not affect the presence or absence of hypertension measured by ABPM.

**TABLE 2:** Evaluation of the presence of hypertension (ABPM) according to demographic variables.

ABPM hypertension		No	Yes			Pearson X^2^
		n	%	n	%	Sig
Marital status	With a partner	172	64.4%	10	71.4%	0.932
	Separated/Divorced	28	10.5%	1	7.1%	
	Single	64	24.0%	3	21.4%	
	Widowed	3	1.1%	0	0.0%	
Social benefit	No	181	70.2%	9	64.3%	0.641
	Yes	77	29.8%	5	35.7%	
Educational level (years of schooling)	12 or more	114	42.9%	4	28.6%	0.559
	5 to 8	28	10.5%	1	7.1%	
	9 to 11	120	45.1%	9	64.3%	
	≤4	4	1.5%	0	0.0%	

Data expressed as absolute (n) and relative (%) frequency. Pearson’s chi-squared test.

**FIGURE 2:**
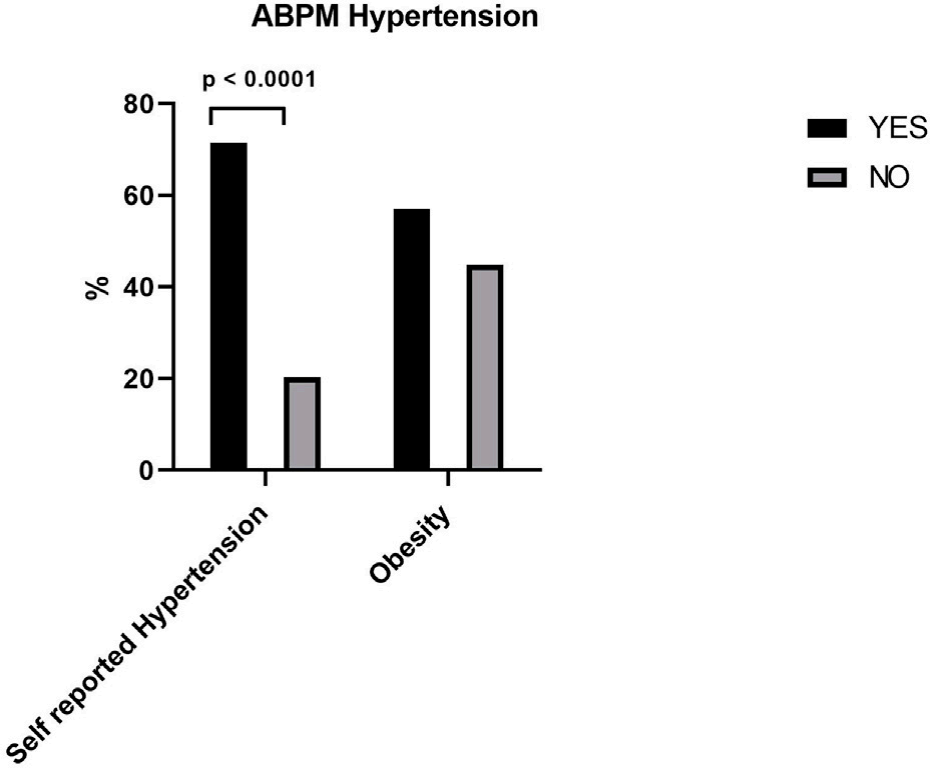
ABPM Hypertension between self reported hypertension and obese women.

Table 3 and Figure 2 shows that there was a statistically significant difference between patients with self-reported hypertension and with hypertension measured by ABPM when asked about the presence of current hypertension. In Table 3 no significant difference was observed between women with hypertension and normotensive measured by ABPM when asked about the presence of high blood glucose or diabetes, or the presence of hypercholesterolemia. ABPM also did not detect hypertension in women with obesity (Figure 2). There was also no difference in the presence of other self-reported comorbidities such as thrombosis, arrhythmia, angina, infarction, stroke, or chronic kidney disease between hypertensive and normotensive women.

**TABLE 3:** Evaluation of the presence of hypertension (ABPM) according to the patient’s self-reported comorbidities.

ABPM hypertension		No		Yes		Pearson X
		n	%	n	%	Sig
High blood glucose/diabetes	No	232	86.9%	13	92.9%	0.515
	Yes	35	13.1%	1	7.1%	
Hypercholesterolemia	No	209	78.6%	11	78.6%	1.000
	Yes	57	21.4%	3	21.4%	
Hypertension	No	213	79.8%	4	28.6%	<0.0001
	Yes	54	20.2%	10	71.4%	
Obesity	No	147	55.1%	6	42.9%	0.372
	Yes	120	44.9%	8	57.1%	
Thrombosis	No	260	97.4%	14	100.0%	0.540
	Yes	7	2.6%	0	0.0%	
Arrhythmia	No	243	92.0%	11	78.6%	0.080
	Yes	21	8.0%	3	21.4%	
Angina	No	246	92.8%	13	92.9%	0.997
	Yes	19	7.2%	1	7.1%	
Infarction	No	265	99.3%	14	100.0%	0.745
	Yes	2	0.7%	0	0.0%	
Stroke	No	266	99.6%	14	100.0%	0.819
	Yes	1	0.4%	0	0.0%	
Kidney disease	No	238	89.1%	11	78.6%	0.225
	Yes	29	10.9%	3	21.4%	

Data expressed as absolute (n) and relative (%) frequency. Pearson’s chi-squared test.

As can be seen in Table 4, there was no difference between patients with hypertension and without hypertension measured by ABPM when asked about the presence of paternal comorbidities. The women were asked whether they had knowledge of the father’s disease, whether their father had high blood glucose or diabetes, and whether they had knowledge of hypercholesterolemia or CVD reported by the father. The women undergoing ABPM were also asked about other paternal comorbidities such as hypertension, obesity, thrombosis, arrhythmia, angina, infarction, stroke, and kidney disease, and no association of the presence of hypertension with these variables was found.

**TABLE 4:** Evaluation of the presence of hypertension (ABPM) according to paternal comorbidities.

ABPM hypertension		No		Yes		Pearson X^2^
		n	%	n	%	Sig
Knowledge of the father’s disease	No	43	16.4%	2	14.3%	0.834
	Yes	219	83.6%	12	85.7%	
Paternal high blood glucose/diabetes	No	151	70.9%	9	75.0%	0.760
	Yes	62	29.1%	3	25.0%	
Hypercholesterolemia reported by the father	No	141	68.4%	7	58.3%	0.466
	Yes	65	31.6%	5	41.7%	
Cardiovascular disease reported by the father	No	165	93.8%	10	90.9%	0.709
	Yes	11	6.3%	1	9.1%	
Paternal hypertension	No	106	50.7%	5	41.7%	0.542
	Yes	103	49.3%	7	58.3%	
Paternal obesity	No	155	72.1%	11	91.7%	0.137
	Yes	60	27.9%	1	8.3%	
Paternal thrombosis	No	196	94.7%	11	91.7%	0.655
	Yes	11	5.3%	1	8.3%	
Paternal arrhythmia	No	182	87.9%	10	83.3%	0.638
	Yes	25	12.1%	2	16.7%	
Paternal angina	No	171	82.2%	8	72.7%	0.428
	Yes	37	17.8%	3	27.3%	
Paternal infarction	No	177	82.7%	9	75.0%	0.496
	Yes	37	17.3%	3	25.0%	
Paternal stroke	No	197	91.2%	11	91.7%	0.956
	Yes	19	8.8%	1	8.3%	
Paternal kidney disease	No	186	88.6%	10	83.3%	0.583
	Yes	24	11.4%	2	16.7%	

Data expressed as absolute (n) and relative (%) frequency. Pearson’s chi-squared test.

Table 5 shows the analysis of the presence of maternal comorbidities reported by the participants according to the presence of hypertension measured by ABPM. Knowledge of maternal diseases such as high blood glucose, diabetes, hypercholesterolemia reported by the mother, CVD reported by the mother, hypertension, obesity, thrombosis, arrhythmia, angina, infarction, stroke or kidney disease did not differ between hypertensive and non-hypertensive women.

**TABLE 5:** Evaluation of the presence of hypertension (ABPM) according to maternal comorbidities.

ABPM hypertension		No		Yes		Pearson X
		n	%	n	%	Sig
Knowledge of the mother’s disease	No	6	2.3%	0	0.0%	0.570
	Yes	260	97.7%	14	100.0%	
Maternal high blood glucose/diabetes	No	173	67.1%	8	57.1%	0.444
	Yes	85	32.9%	6	42.9%	
Hypercholesterolemia reported by the mother	No	136	54.0%	10	71.4%	0.201
	Yes	116	46.0%	4	28.6%	
Cardiovascular disease reported by the mother	No	80	44.9%	8	72.7%	0.073
	Yes	98	55.1%	3	27.3%	
Maternal hypertension	No	102	39.5%	4	28.6%	0.413
	Yes	156	60.5%	10	71.4%	
Maternal obesity	No	148	57.1%	10	71.4%	0.292
	Yes	111	42.9%	4	28.6%	
Maternal thrombosis	No	239	94.1%	14	100.0%	0.349
	Yes	15	5.9%	0	0.0%	
Maternal arrhythmia	No	201	79.1%	13	92.9%	0.213
	Yes	53	20.9%	1	7.1%	
Maternal angina	No	199	77.7%	11	84.6%	0.559
	Yes	57	22.3%	2	15.4%	
Maternal infarction	No	229	89.1%	13	92.9%	0.658
	Yes	28	10.9%	1	7.1%	
Maternal stroke	No	243	94.2%	12	85.7%	0.202
	Yes	15	5.8%	2	14.3%	
Maternal kidney disease	No	229	90.2%	14	100.0%	0.218
	Yes	25	9.8%	0	0.0%	

Data expressed as absolute (n) and relative (%) frequency. Pearson’s chi-squared test.

**FIGURE 3:**
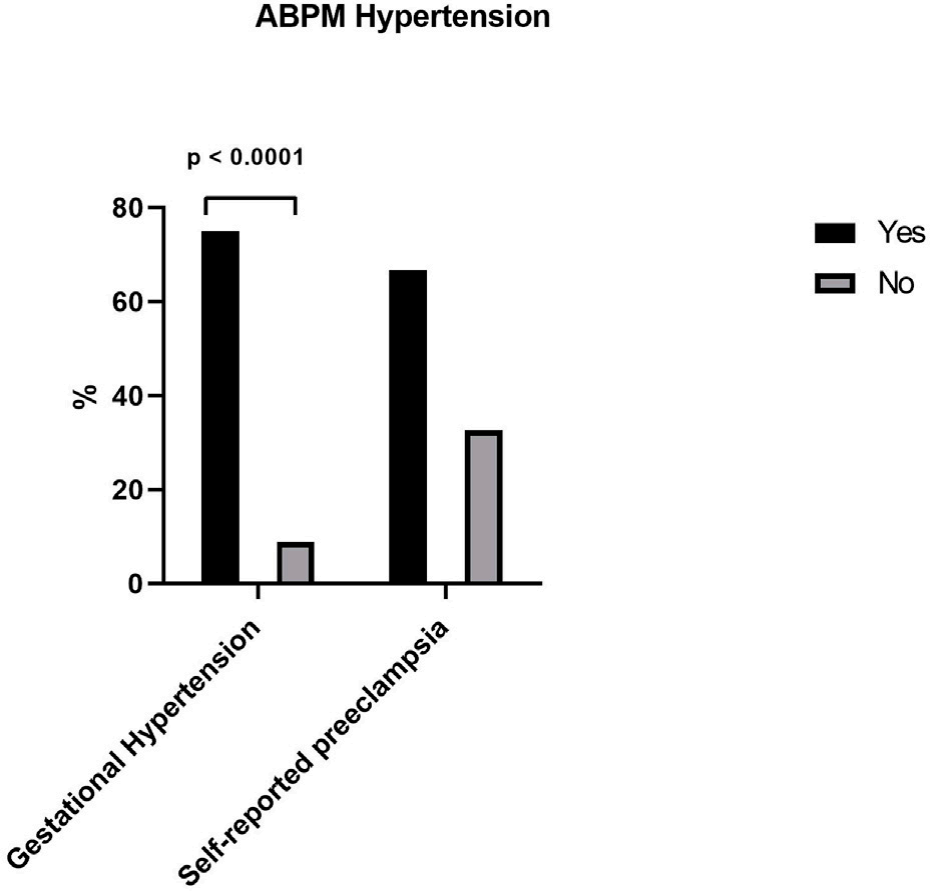
ABPM Hypertension between gestational hypertension and self reported hypertension.

When we evaluated the presence of hypertensive disorders of pregnancy, we found a significant difference in patients classified as hypertensive by ABPM when asked about gestational hypertension (*p* < 0.0001) and we found no presence of hypertension measured by ABPM when the patient was asked about having preeclampsia (Figure 3).

Table 6 shows the evaluation of the presence of hypertension measured by ABPM according to anthropometric parameters (waist circumference, neck circumference, weight, height, BMI, weight-to-height ratio and no significant difference was observed between hypertensive and non-hypertensive women. As can be seen in Table 7, there was no significant difference in markers of CVD (serum triglycerides, cholesterol, LDL, HDL, creatinine, glucose, C-reactive protein, homocysteine, basal insulin, and glycated hemoglobin) between hypertensive and non-hypertensive women based on ABPM. Also in Table 7, there was no difference in pulse wave velocity between hypertensive and non-hypertensive women.

**TABLE 6:** Evaluation of the presence of hypertension (ABPM) according to anthropometric measurements.

ABPM hypertension	No			Yes			Mann-whitney
	Median	25%	75%	Median	25%	75%	Sig
Waist circumference	87.0	79.0	98.0	93.0	85.0	107.0	0.113
Neck circumference	35.0	33.0	37.0	35.8	34.0	38.0	0.094
Weight	71.8	62.4	87.2	77.1	65.5	97.3	0.361
Height	162.0	158.0	166.0	160.8	157.0	166.0	0.756
BMI	27.6	24.0	33.2	29.1	26.0	33.3	0.340
Waist-height ratio	0.54	0.49	0.61	0.54	0.53	0.69	0.112

Data expressed as median and percentile (25%–75%). Mann-Whitney test.

**TABLE 7:** Evaluation of the presence of hypertension (ABPM) according to serum levels of cardiovascular disease markers.

ABPM hypertension	No			Yes			Mann-whitney
	Median	25%	75%	Median	25%	75%	Sig
Triglycerides	108.0	76.0	153.0	150.5	98.0	197.0	0.100
Cholesterol	174.5	152.0	197.0	195.5	161.0	212.0	0.221
LDL	100.0	82.0	123.5	103.5	85.0	133.0	0.710
HDL	46.9	40.2	55.1	46.4	38.2	61.1	0.752
Creatinine	0.80	0.71	0.88	0.84	0.69	0.93	0.632
Glucose	88.0	79.0	97.0	85.4	77.0	100.1	0.649
C-reactive protein	0.31	0.12	0.66	0.40	0.21	0.55	0.389
Homocysteine	7.7	6.8	9.3	7.7	6.5	9.3	0.806
Basal insulin	27.2	14.7	53.0	33.6	29.0	43.0	0.519
Glycated hemoglobin	5.3	5.0	5.6	5.3	5.1	5.4	0.935
PWV ^(a)^	7.0	6.2	7.8	7.9	6.6	8.5	0.130

(a) Pulse wave velocity; Data expressed as median and percentile (25%–75%). Mann-Whitney test.

## Discussion

The main findings of the present study were: that white women had hypertension detected by the ABPM; women who reported that they were aware of being hypertensive in the interview when they were measured by the ABPM had their hypertension demonstrated by blood pressure data; gestational hypertension measured by the ABPM presented hypertensive.

Two approaches were used in this study to assess the presence of arterial hypertension: one was through a questionnaire, the women answered if they knew if they were hypertensive and answered positively and the other was through quantitative measures such as measured by ABPM confirming hypertension. Hypertension was reported during the interview by 19.1% of the women, and the prevalence of hypertensive among women measure by ABPM was 4.98% In addition, 4.95% of the participants reported preeclampsia during a pregnancy.

In our study, we demonstrated a statistically significant difference in white skin color in which women were detected as having hypertension by ABPM while other ethnicities such as black and mixed women did not have this difference. The studies by Mendes et al. (2018) and Paula et al. (2020) discuss the differences between hypertensive and normotensive people when the topic is ethnicity or race. Other demographic characteristics such as marital status and educational level did not show a statistical difference showing the presence or absence of hypertension when measured by the ABPM.

Regarding cardiovascular comorbidities, our study found no significant associations between hypertension measured by ABPM and diabetes, obesity, infarction, stroke, chronic kidney disease, or other comorbidities. Comparing these data with the literature, it is known that hypertension is associated with several risk factors for cardiovascular diseases (CVD), including lipid disorders such as high levels of cholesterol and triglycerides, obesity, and tobacco and alcohol consumption. It is also known that the presence of more than one risk factor for CVD such as heart attack, chronic kidney disease and stroke are increased in patients with hypertension (Levine et al., 2018; Unger et al., 2020). In our study, due to the small number of women, we did not find this association. Regarding paternal and maternal comorbidities, our study did not find hypertension to be associated with these comorbidities It is also known that paternal and maternal comorbidities influence the presence of hypertension, another result different from the literature in our study (Levine et al., 2018; Unger et al., 2020).

Our study also assessed the presence of hypertension measured by ABPM according to with median measures of anthropometric parameters, including abdominal circumference, neck circumference, weight, height, BMI, waist-to-height ratio. There was no significant difference in these parameters, i.e., the anthropometric measurements, always considering that the anthropometric parameters are with median values, of the participants are not related to the presence of hypertension. In the literature we find that we found that in the study by Cassani et al. (2009) only waist circumference was associated with hypertension detected by conventional blood pressure measurement, while other anthropometric parameters were not associated with hypertension, as observed in our study with median measurements. Schommer et al. (2014) also reported an association of waist circumference with hypertension in 10- to 18-year-old adolescents (Cassani et al., 2009; Schommer et al., 2014).

When hypertensive patients based on ABPM were evaluated in our study regarding the presence of hypertensive disorders of pregnancy, there was a significant difference when these patients were asked about the presence of gestational hypertension, women who reported gestational hypertension were hypertensive based on ABPM. However, there was no relationship between the presence of self-reported preeclampsia and hypertension measured by ABPM. Metoki et al. (2022) recommend ABPM in hypertensive and overweight pregnant women. In a review article, Bello et al. (2018) recommend the use of ABPM in pregnant women as a tool to guide the diagnosis and management of hypertensive disorders of pregnancy (Bello et al., 2018; Metoki et al., 2022).

We also observed no significant differences in serum cardiovascular markers such as LDL, HDL, C-reactive protein, triglycerides or cholesterol between women with hypertension measured by ABPM and non-hypertensive women, also in this case the evaluation of cardiovascular markers was with average values (Babkowski et al., 2021). Kidambi et al. (2020) reported that, in African Americans, 24-h ABPM is a limited predictor of CVD.

The pulse wave velocity measure is a measure of arterial stiffness and there was no relationship with the detections of the presence of hypertension in women, probably due to the low mean age of women.

Our study has several strengths such as the assessment of many different variables–from demographic variables such as participants’ comorbidities and paternal and maternal comorbidities of the participants to anthropometric and serum dosage variables–to determine the presence of hypertension measured by the ABPM. This study also serves to reinforce the use of the ABPM to measure hypertension in women aged close to 40 years to encourage future research and cohorts to investigate hypertension in this age group in women. However, among the limitations of the study are that the women who underwent evaluation by ABPM were relatively few (*n* = 281), that is, the acceptance of the placement of the ABPM voluntarily by women was low, with only 281 accepting to do this specific evaluation. Of the total of 929 women who participated in the cohort. Another limitation is that these women were evaluated at a relatively young age (38–39 years) for the development of cardiovascular diseases, it is consolidated in the literature that cardiovascular diseases, including hypertension, appear more frequently in older age groups, more specifically over 50 and over 60 years.

## Conclusion

We conclude that ethnicity, self-reported hypertension, and the presence of hypertension during pregnancy are associated with arterial hypertension measured by ABPM. We did not find this association for the other parameters analyzed in part because the women analyzed in the study were aged 39–39 years. However, the identification of risk factors in women by ABPM is important for preventing future CVD, reducing morbidity and mortality and improving quality of life and longevity.

## Data Availability

The raw data supporting the conclusions of this article will be made available by the authors, without undue reservation.
